# Choroid metastases revealing primary clear cell adenocarcinoma of the lung effectively treated with cisplatin and pemetrexed: a case report

**DOI:** 10.1186/1752-1947-7-267

**Published:** 2013-12-11

**Authors:** Yueh-Shih Chang, Ling Yeung, Liang-Che Chang, Jen-Seng Huang, Kun-Yun Yeh

**Affiliations:** 1 Division of Hemato-oncology, Department of Internal Medicine, Chang Gung Memorial Hospital, Keelung & Chang Gung University, College of Medicine, 222 Maijin Road, Keelung, Taiwan; 2 Department of Ophthalmology, Chang Gung Memorial Hospital, Keelung & Chang Gung University, College of Medicine, Keelung, Taiwan; 3 Department of Pathology, Chang Gung Memorial Hospital, Keelung & Chang Gung University, College of Medicine, Keelung, Taiwan

## Abstract

**Introduction:**

The aim of the present report was to draw the attention of oncologists to the importance of prompt diagnosis of primary clear cell adenocarcinoma of the lung, which allows early initiation of treatment to maintain quality of life.

**Case presentation:**

A 63-year-old Chinese woman initially presented to our facility with multifocal bilateral choroid metastatic lesions that were found to originate from a primary clear cell adenocarcinoma of the lung (T2bN2M1b, stage IV). A thorough ophthalmologic evaluation, study of our patient’s history, imaging studies and comprehensive immunohistochemical staining tests led to the diagnosis of this rare lung tumor.

**Conclusions:**

Although this uncommon cancer is unfortunately already at a late stage when choroid metastases develop, systemic chemotherapy alone is sufficient to preserve vision and gain control over the disease.

## Introduction

Choroid metastasis (CM) is the most common intra-ocular neoplasm in adults, and the most common sites for the primary tumors are the breast and lung [1-4]. The presence of such metastases suggest hematogenous spread, an advanced stage of cancer therefore giving a poor prognosis [5]. The prompt detection of CM is of great significance as, with consideration of the performance status of the patient and location of the primary tumor, it can lead to early implementation of appropriate therapeutic management to improve disease control.

Multiple foci and bilateral involvement are important clinical presentations of CM that can lead to the correct diagnosis of the primary malignancy [1]. Clear cell adenocarcinoma of the lung with an abundant clear cell component is extremely rare, and the associated biological behavior and treatment outcomes for this illness are unknown. Here, we present a case of primary clear cell adenocarcinoma of the lung with multifocal bilateral CM as the initial presentation in an otherwise healthy woman who had a significant and durable response to systemic chemotherapy using cisplatin and pemetrexed.

## Case presentation

**Figure 1:**
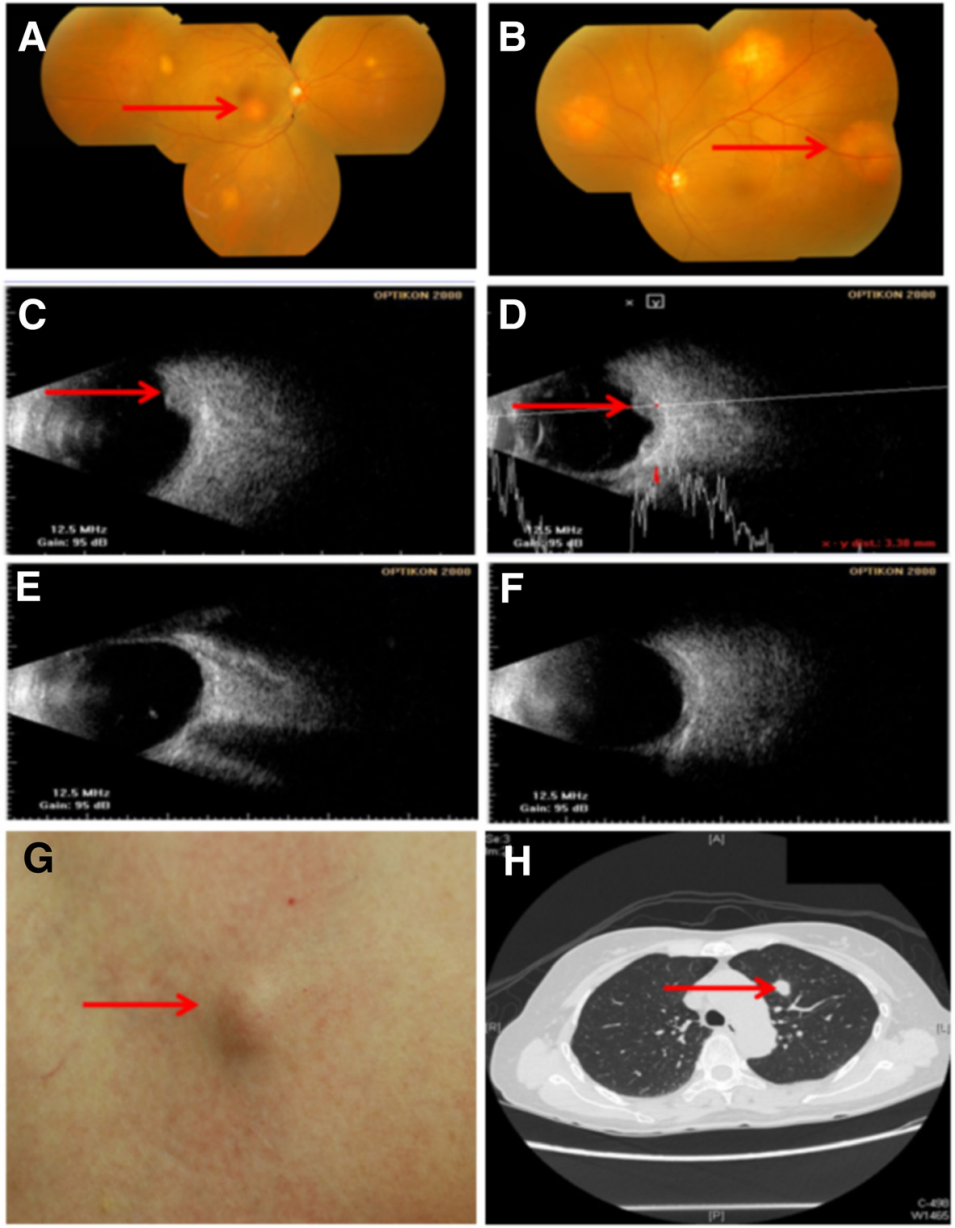
**Choroid and skin metastases of primary clear cell adenocarcinoma of lung. (A-F)** Ophthalmology images; **(A,C,E)** right eye, **(B,D,F)** left eye. **(A,B)** Fundus appearance before treatment (arrows point to lesions); **(C,D)** ultrasound scan of the same eyes as in **(A,B)** before treatment (arrows point to lesions); **(E,F)** lesion resolution by ultrasound scan of the same eyes as in **(A,B)** after treatment. **(G)** Appearance of skin metastasis (arrow). **(H)** Chest computed tomography scan showing left upper lobe mass (1.3cm in diameter) (arrow).

**Figure 2:**
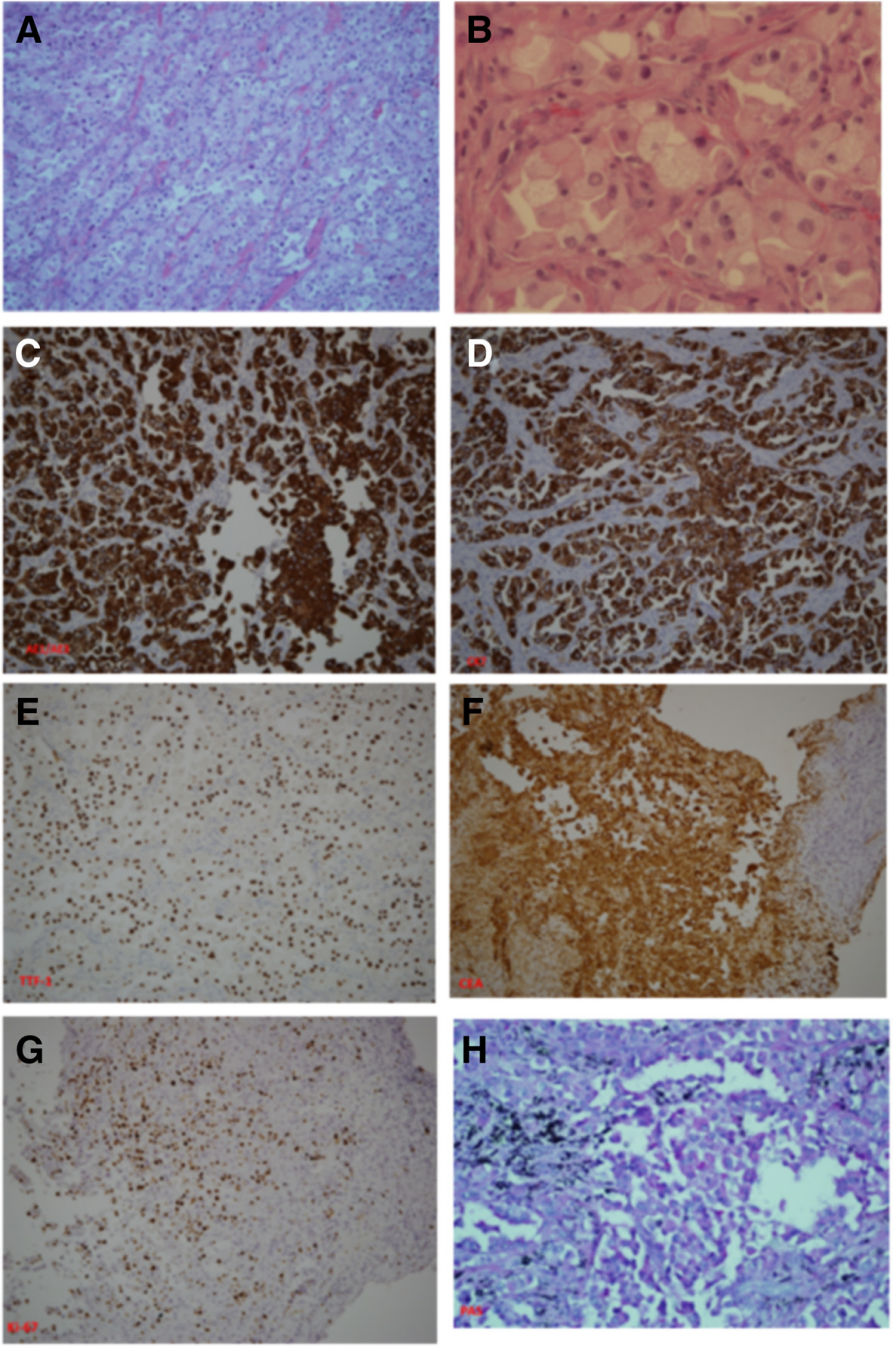
**Histopathological findings of primary clear cell adenocarcinoma of the lung.** Hematoxylin and eosin staining showed that the main tumor was infiltrated by pleomorphic clear tumor cells with foamy cytoplasm and distinct nucleoli (**(A)**, 100×; **(B)** 400×). Positive immunohistochemical staining results included pancytokeratin AE1/AE3 **(C)**, cytokeratin 7 **(D)**, thyroid transcription factor 1 **(E)**, Ki-67 **(G)**, carcinoembryonic antigen **(F)** and periodic acid Schiff **(H)**. Negative immunohistochemical stainings included HMB-45, Hep-par-1, transcription factor E3, α-inhibin, S-100 and CDX2 (data not shown).

A 63-year-old Chinese woman presented to our facility with a history of bilateral photopsia and blurred vision for the last two months. She had no history of seizure, vomiting, head injury, or exposure to medications that could cause such effects. A thorough ophthalmological and systemic examination was carried out. An ocular examination showed best corrected visual acuity of 20/100 in the right eye and 2/80 in the left eye. A fundoscopic examination revealed the presence of multiple choroidal masses in both eyes (Figure 1A,B). An ultrasonographic evaluation of the eye demonstrated elevated choroidal masses in both eyes with a maximal elevation of 3.2mm (Figure 1C,D). A physical examination showed skin nodules over the chest and abdominal areas. The pathology of the skin nodules revealed metastatic clear cell adenocarcinoma of unknown origin. Computerized tomography of the chest, abdomen and pelvic area was performed. A mass in the left upper lobe of the lung with several enlarged mediastinal lymph nodes was detected. Moreover, skin nodules were found on the chest and abdominal walls, and a left adrenal nodule was also present (Figure 1G,H). A bone scan revealed multiple metastases. Our patient then underwent a video-assisted thoracic surgical (VATS) biopsy to obtain adequate material to establish a pathological diagnosis. Histologically, the tumors from lung parenchyma, pleura and mediastinal lymph nodes were infiltrated by core-like or abortive glandular structures that consisted of pleomorphic clear tumor cells with foamy cytoplasm and distinct nucleoli (Figure 2A,B). Immunohistochemical (IHC) staining test results showed that the tumor cells were positive for pancytokeratin (AE1/AE3) (Figure 2C), cytokeratin 7 (CK-7) (Figure 2D), thyroid transcription factor 1 (TTF-1) (Figure 2E) and carcinoembryonic antigen (CEA) (Figure 2F). The results for Ki-67 staining displayed a proliferative index of approximately 45 to 50 percent (Figure 2G). Results of a histochemical stain showed tumor cells positively stained by periodic acid Schiff (PAS) and PAS with diastase indicated the presence of glycogen (Figure 2H) In contrast, the tumor cells tested negative for CK-5/6, CK-20, vimentin, thyroglobulin, CD10, CDX2, epithelial membrane antigen (EMA), transcription factor E3 (TFE-3), α-inhibin, Hep-par-1, glypican-3, p63 and HMB-45 (data not shown). The clinical and pathological features of our patient’s case were compatible with a clear cell adenocarcinoma of the lung (T2bN2M1b, stage IV, according to the American Joint Committee on Cancer (AJCC) cancer staging guide, seventh edition).

Systemic chemotherapy with pemetrexed (500mg/m^2^, every three weeks) and cisplatin (75mg/m^2^, every three weeks) was initiated. After cycle six of chemotherapy, a fundoscopic examination showed near complete resolution of the choroidal masses, coinciding with improvement in her vision (Figure 1E,F). Although her cancer status outside the orbit remained stable, she had a good quality of life with no chemotherapy maintenance for over 24 months at the time of this report.

## Discussion

Adenocarcinoma with cells that are filled with clear cytoplasm and that are the major component of a lung tumor is defined as primary clear cell adenocarcinoma of the lung according to World Health Organization classification guidelines [6]. Because such pulmonary adenocarcinomas are very small in number, and the cases reported in the literature have shown varied biological natures and clinical presentations [7], the appropriate diagnostic procedures may help to establish an accurate diagnosis*.* Firstly, it is so histologically heterogeneous for pulmonary adenocarcinomas that we used video-assisted thoracoscopy to acquire enough tumor tissue, even though a fine-needle aspiration biopsy may enable early diagnosis and hence the initiation of immediate appropriate palliative therapy. Secondly, comprehensive IHC staining studies are required. The tumor tissue from our patient showed monotonous clear cells arranged in a glandular pattern, positive immunoreaction results for AE1/AE3, CK-7, TTF-1, CEA, Ki-67, and positive PAS staining for the existence of glycogen in clear cell cytoplasm. Thus, our patient’s tumor met the criteria for primary clear cell adenocarcinoma of the lung. Finally, our patient’s tumor was distinguished from tumors with clear cell cytoplasm in any organs. Our patient’s tumor was different from renal clear cell carcinoma because there was no tumor found on imaging studies and the tumor cells were negative for CD10 staining. It was not a benign clear cell sugar tumor because it was malignant and the tumor cells were negative for HMB-45. Our patient’s case was not one of clear cell squamous cell carcinoma in terms of histological features and negative staining for p63. It was obviously not a pulmonary blastoma or fetal adenocarcinoma because no fetal features were present. Our patient’s tumor was not a metastatic thyroid papillary clear cell carcinoma because there was no tumor found in the thyroid glands and no expression of thyroglobulin. Our patient’s case was not one of clear cell sarcoma, malignant melanoma, sex-cord tumor and metastatic hepatocellular carcinoma, alveolar soft part tumor and metastatic carcinomas of the gastrointestinal tract because there was negative staining for HMB-45, S-100, α-inhibin, Hep-par-1, glypican-3, CDX2, TFE-3 and vimentin. Taken as whole, our patient’s clinical presentation, imaging study results and thorough investigation of pathological features and IHC stainings were sufficient to confirm the diagnosis of her tumor as a primary clear cell adenocarcinoma of the lung with multifocal and bilateral CM, even without a choroid biopsy.

The incidence of CM for lung cancer is reported to be no more than 7 percent [8]. In such a condition, the lung cancer is already at a late stage and dissemination of the disease has occurred; the median survival after diagnosing symptomatic CM is 4.3 to 7.4 months [9]. Hence, the goals of treatment are to improve quality of life and preserve vision, which can be achieved by either systemic chemotherapy or target therapy based upon the performance status and the histologic subtype. It has been demonstrated that inhibition of the epidermal growth receptor (EGFR) pathway with tyrosine kinase inhibitors (TKIs) provides effective treatment and improved tolerability for non-small-cell lung cancers, especially for a subset of female patients of East Asian origin who are never smokers and have an adenocarcinoma histology, and who have high prevalence of sensitive activating EGFR mutations (deletion in exon 19 or point mutation of L858 in exon 21) [10]. However, TKIs were not considered since the tumor specimen from our patient failed to show the aforementioned EGFR mutation.

It has been reported that systemic chemotherapy is efficacious for choroid metastases from non-small-cell lung cancer [11,12]. In our patient’s case, standard platinum doublet chemotherapy was used as the initial and primary treatment because our patient had other concurrent sites of metastatic disease that required simultaneous attention. Further, cisplatin plus pemetrexed has shown survival benefits in pulmonary adenocarcinoma [13]. Although the tumor remained stable, our patient gained visual improvement and acceptable disease control via this platinum doublet chemotherapy. Taken together, these findings suggest the choroid may be no longer considered as a ‘sanctuary site’ to chemotherapy, and systemic chemotherapy alone is a useful therapeutic option for CM of lung cancer.

The prognosis and treatment choices for primary clear cell adenocarcinoma of the lung may resemble those for non-small-cell lung cancer. Because the disease is rare and glycogen-rich clear cells display an unusual histology, the prognosis and treatment outcome may be more complicated. Even though a patient may initially present with multiple metastases including the choroidal type, as in our patient’s case, immediate initiation of histologically-adapted chemotherapy may yield clinical benefits and improvements in quality of life. To the best of our knowledge this is the first report demonstrating that the efficacy and safety of systemic doublet chemotherapy with cisplatin plus pemetrexed for choroidal metastasis of primary clear cell adenocarcinoma of the lung.

## Conclusions

In summary, we present a rare occurrence of clear cell adenocarcinoma of the lung that needed to be confirmed and differentiated from clear cell cancer from other organs by systemic investigation and intensive IHC staining tests. CM may be the initial sign of this rare tumor and the ophthalmologic diagnosis may lead to the discovery of the systemic affliction, albeit unfortunately at a late stage of the disease. If compatible with the patient’s general status, appropriate chemotherapy may be an efficacious mode of treatment in advanced stages of this rare and metastatic lung cancer.

## Consent

Written informed consent was obtained from the patient for publication of this manuscript and any accompanying images. A copy of the written consent is available for review by the Editor-in-Chief of this journal.

## Competing interests

The authors declare they have no conflicts of interest in employment (other than primary affiliations), commercial grants, other commercial research support, ownership interests, consultant/advisory board interests and honoraria from speakers’ bureaus.

## Authors’ contributions

YL provided all ophthalmological examination data. CL-C performed all pathological examinations and interpreted these results. HJ-S shared his previous treatment experience. YK-Y is the corresponding author, was our patient’s attending doctor, takes responsibility for integrating all information and made the final treatment decisions. CY-S was the major contributor to writing the manuscript. All authors read and approved the final manuscript.
